# Inflammasomes at the crossroads of traumatic brain injury and post-traumatic epilepsy

**DOI:** 10.1186/s12974-024-03167-8

**Published:** 2024-07-16

**Authors:** Mohit Javalgekar, Bianca Jupp, Lucy Vivash, Terence J. O’Brien, David K. Wright, Nigel C. Jones, Idrish Ali

**Affiliations:** 1 The Department of Neuroscience, School of Translational Medicine, Monash University, 99, Commercial Road, Melbourne, Australia; 2 Department of Neurology, The Alfred Hospital, 99 commercial road, Melbourne, Australia; 3 The University of Melbourne, Parkville, Australia

**Keywords:** Post-traumatic epilepsy, Traumatic brain injury, Inflammasome, Microglia, NLRP3

## Abstract

Post-traumatic epilepsy (PTE) is one of the most debilitating consequences of traumatic brain injury (TBI) and is one of the most drug-resistant forms of epilepsy. Novel therapeutic treatment options are an urgent unmet clinical need. The current focus in healthcare has been shifting to disease prevention, rather than treatment, though, not much progress has been made due to a limited understanding of the disease pathogenesis. Neuroinflammation has been implicated in the pathophysiology of traumatic brain injury and may impact neurological sequelae following TBI including functional behavior and post-traumatic epilepsy development. Inflammasome signaling is one of the major components of the neuroinflammatory response, which is increasingly being explored for its contribution to the epileptogenic mechanisms and a novel therapeutic target against epilepsy. This review discusses the role of inflammasomes as a possible connecting link between TBI and PTE with a particular focus on clinical and preclinical evidence of therapeutic inflammasome targeting and its downstream effector molecules for their contribution to epileptogenesis. Finally, we also discuss emerging evidence indicating the potential of evaluating inflammasome proteins in biofluids and the brain by non-invasive neuroimaging, as potential biomarkers for predicting PTE development.

## Neuroinflammation in epilepsy: a role for inflammasome signaling?

Traumatic brain injury (TBI) occurs after a physical trauma to the head or neck, leading to functional and pathological changes in the brain [1]. In addition to behavioral and psychiatric disorders which are frequent consequences, one of the most serious chronic sequelae of TBI is the development of post-traumatic epilepsy (PTE) [2, 3]. Epilepsy is a neurological disease diagnosed by the recurrence of spontaneous epileptic seizures, and PTE specifically refers to epilepsy caused by a preceding TBI. Epileptogenesis refers to the neurobiological processes that result in the transformation of a healthy brain into an epileptic brain through complex molecular and functional aberrations that can be triggered by brain insults such as TBI [4]. The location and severity of TBIs are physical characteristics that have a significant impact on PTE incidence. Increased risk of PTE is linked to lesions in the temporal lobe and penetrating injuries in the temporal and parietal lobes [5]. For PTE, epileptogenesis often occurs months to years after the precipitating injury, and this delay period is referred to as the silent, or latent period [6, 7]. During this period, active but currently undetectable, molecular, inflammatory, and structural brain changes occur which ultimately culminate in the emergence of spontaneous seizures and epilepsy. This latent period between TBI and the onset of seizures provides a window for therapeutic anti-epileptogenic intervention. However, the community has yet to identify effective interventions that can interrupt this process of post-traumatic epileptogenesis in humans. In addition, initiating preventive therapies during this period remains conceptually challenging because of the lack of predictive biomarkers that can identify TBI patients at high risk of developing epilepsy [8–10]. A greater understanding of the pathophysiological mechanisms driving post-traumatic epileptogenesis may enable the identification of specific biomarkers of PTE, as well as disease-modifying therapeutics.

In recent years, neuroinflammation has emerged as a potential mechanism driving secondary brain injury. Based on the severity of initial trauma, the extent of neuroinflammation greatly enhances the risk of PTE development [11]. Neuroinflammation following TBI involves the activation and migration of resident glial cells to the site of injury, and the release of inflammatory mediators such as cytokines and chemokines [12]. The acute phase of neuroinflammation provides neuroprotection by limiting the spread of damage and clearing cellular debris [13]. However, the ensuing chronic activation of glial cells may lead to further damage to the surrounding tissue around the primary injury area characterized by ionic imbalance, excitatory amino acid release, calcium overload, generation of reactive oxygen species(ROS), disruption of the cell membrane, vascular rupture, and ultimately cell death [14]. The end products derived from dead or damaged cells constitute Danger-Associated Molecular Patterns (DAMPs) leading to further stimulation of microglia and astrocytes and secretion of cytokines, chemokines, adhesion molecules and transcriptional upregulation of pro-inflammatory factors like NF-κB [15]. In addition, the integrity of the blood-brain barrier is changed as a direct result of TBI and cytokine signalling leading to the recruitment of peripheral immune cells such as blood-derived leucocytes, macrophages and serum proteins in brain parenchyma [16]. Altered BBB permeability has been shown to induce neuronal hyperexcitability possibly via TGF-β receptor-mediated astrocytic serum albumin uptake and extracellular potassium accumulation [17]. Other studies have shown that BBB disruption causes epileptiform discharges in rat somatosensory cortex [18] and a direct correlation between the seizure burden and the extent of BBB damage in TBI patients and preclinical TBI model [19]. Furthermore, the introduction of serum protein in the brain through leaky BBB could initiate alternate pathways like complement activation while peripheral immune cells such as neutrophils along with the activated glial cells exacerbate the inflammatory cascade [20, 21]. This inflammatory continuum can ultimately result in progressive neuronal loss and worsening of injury outcomes [22].

Neuroinflammation following an experimental brain injury can be sustained for at least six months [23]. Persistent neuroinflammation can also influence several brain processes implicated in epileptogenesis, including neuronal plasticity, neuronal circuit reorganization, alterations in excitatory and inhibitory neurotransmitter receptors and ion channels, and changes in the regulation of neuronal transmission [11].

One neuroinflammatory component that may be particularly relevant to PTE is the inflammasome signaling complex. Inflammasomes are protein complexes generated in neuroinflammatory environments that are involved in the activation and release of caspase-1 and inflammatory cytokines [24]. Literature has independently shown the significant contribution of inflammasomes in TBI phenotypes and seizure generation [25, 26]. In this review, we bring this literature together to explore the possible contribution of inflammasomes to PTE. We first describe the mechanisms of assembly of inflammasome complexes, discuss inflammasome signaling following TBI and the role of key inflammasome mediators in epileptogenesis. We also summarize clinical and preclinical examples of therapeutic inflammasome targeting and explore the potential of inflammasomes as biomarkers of PTE. For this narrative review, we searched the PubMed database using combinations of the following search criteria: inflammasome, activation and assembly, epileptogenesis, TBI, molecular mechanisms, therapeutic intervention, and biomarker. For the Tables that summarize this literature, we included all original studies that assessed inflammasome activation in TBI or epilepsy in both human subjects and model systems. We excluded all non-original articles such as reviews, letters, books, and conference abstracts.

## Assembly of the inflammasome complex

**Fig. 1:**
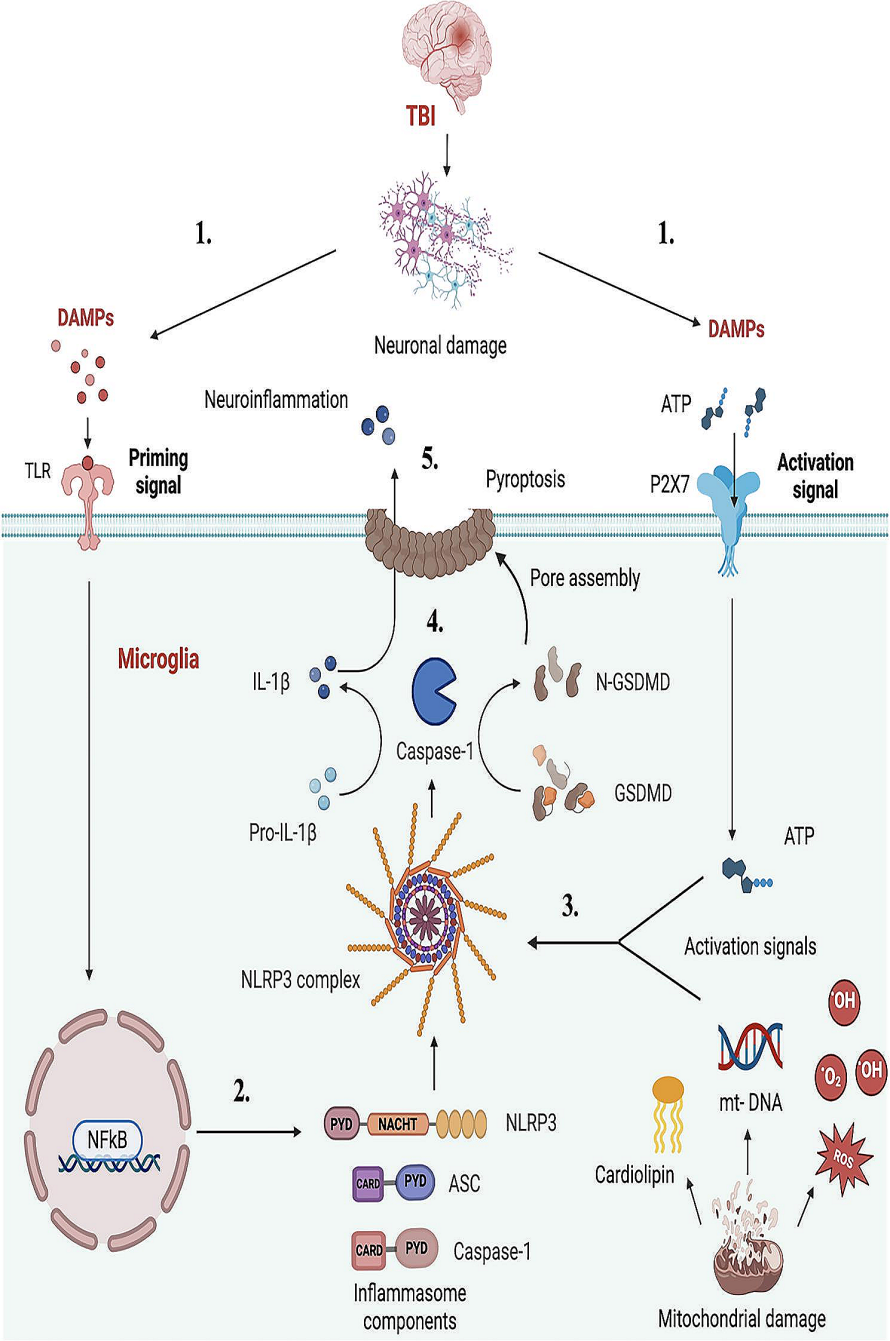
NLRP3 inflammasome activation following TBI. (1) TBI causes neuronal damage resulting in DAMP generation. (2) DAMPs initiate TLR-mediated NFκB activation and subsequent expression of the inflammasome gene. (3) Secondary activation signals from DAMPs such as extracellular ATP, and damaged mitochondrial components like ROS, DNA, and cardiolipin lead to inflammasome assembly, activation, and maturation of caspase-1. (4) Synthesis of mature IL-1β and Gasdermin D by activated caspase-1. 6) Plasma membrane rupture causing pyroptotic cell death by Gasdermin D and release of IL-1β in extracellular space resulting in persistent inflammatory response

Inflammasomes are a series of cytosolic multimeric protein complexes made up of sensory receptor and adaptor proteins that, when aligned, activate caspase-1 to stimulate the maturation and release of pro-inflammatory cytokines [24]. Inflammasome complex formation is well-characterized (Fig. 1) and is integral to the innate immune response. The formation is initiated via the activation of pattern recognition receptors (PRRs) by DAMPs such as ATP, mitochondrial DNA and histones released from dead or damaged neurons. The PRRs of the innate immune system are categorized into five categories: Absent in melanoma-2 (AIM2)-like receptors (ALRs), toll-like receptors (TLRs), nucleotide oligomerization domain (NOD)-like receptors (NLRs), retinoic acid-inducible gene-I (RIG-I)-like receptors (RLRs), and C-type lectin receptors (CLRs) [27]. These receptors are typically expressed on the surface of microglia, the resident brain immune cells, along with peripheral macrophages, monocytes, neutrophils, and other cells involved in the primary immune defense [24]. A subset of these PRRs is the intracellular NLR (Nod-like receptor) family consisting of a central nucleotide-binding and oligomerization (NACHT) domain surrounded by C-terminal leucine-rich repeats (LRRs) and an N-terminal caspase recruitment domain (CARD) or pyrin (PYD) domain. The LRRs act as ligand detectors while CARD and PYD domains interact with adaptor protein during the inflammasome assembly. Apoptosis-associated Speck-like protein containing a CARD (ASC) is the adaptor protein containing the PYD and CARD domain [28]. To date, the NLR family of inflammasomes is the most extensively studied and members such as NLRP1 and NLRP3 complexes have been implicated in several conditions associated with neuroinflammation, such as Alzheimer’s disease, multiple sclerosis, and TBI [29]. The mechanism of inflammasome assembly, activation and downstream signaling has been illustrated with NLRP3 activation following TBI as an example in Fig. 1.

Upon detecting the danger signals, there is activation of receptor proteins (NLRs) and the recruitment of the adaptor protein ASC. ASC bridges with the upstream sensor molecule via a homotypic PYD-PYD or CARD-CARD interaction to form a sensor-adaptor complex [30]. Following this initial step, the next stage comprises of recruitment of pro-caspase-1 by the adaptor protein ASC via CARD interaction completing the inflammasome complex formation [31]. Bioactive caspase-1 then activates inflammatory cytokines like Interleukin 1β (IL-1β) and Interleukin 18 (IL-18) by cleavage of their inactive precursor forms pro-IL-1β and pro-IL-18. These activated cytokines in turn can alter the cellular environment through various mechanisms ranging from disruption of intracellular ionic balance to altered blood-brain barrier (BBB) permeability which may stimulate further inflammatory cascade [32]. Unlike other inflammasome complexes, the mechanism of NLRP3 activation is a two-step step process. A priming signal induced by the PRR such as TLR induces NF-κB mediated transcriptional upregulation of pro-IL-1β and NLRP3 proteins. A secondary signal such as P2 purinoreceptor 7 (P2 × 7) signaling by extracellular ATP or the damaged mitochondrial components such as ROS, DNA and cardiolipin causes the assembly of individual inflammasome components to form a mature NLRP3 complex [33]. Some inflammasomes like NLRP1 and NLRC4 can activate caspase-1 by directly interacting with the CARD domain of caspase-1 without engaging the adaptor protein ASC [34, 35].

Caspase-1 also induces a characteristic cell death called pyroptosis by activating the pore-forming protein Gasdermin D [24, 36]. This causes leakage of cytosolic contents through pores formed on the plasma membrane, further aggravating inflammation (described further later). Following pyroptotic cell death, ASC oligomers continue to process IL-1β extracellularly, as well as stimulate macrophages for further IL-1β release [37]. In addition, an alternate pathway of inflammasome activation, termed non-canonical activation, causes the activation and release of caspase-11 in murine or caspase-4/5 in human cells [38, 39]. As was previously indicated, the connection between inflammasome activity and several CNS disorders has piqued interest in how they may affect the neuroinflammatory response after TBI and post-trauma sequelae like epilepsy and is discussed in detail in the following sections.

## Inflammasomes are activated after TBI

Inflammasomes have been heavily implicated in chronic neuroinflammation associated with TBI [40]. For example, in the clinical setting, increased expression of inflammasome complex proteins NLRP1, NLRP3 and ASC, as well as the matured end products active caspase-1, IL-1β and IL-18 have been consistently reported in the CSF of adult TBI patients [41–43]. In keeping with findings from the adult population, enhanced NLRP3 expression is also observed in the CSF of children and infants with severe TBI [44]. Recent clinical trials targeting inflammasomes in systemic inflammatory conditions, such as COVID-19, gout, heart failure and autoimmune diseases have reported improved outcomes and satisfactory safety and efficacy profiles [25]. Studies targeting inflammasomes in CNS pathologies such as in TBI patients have not been reported yet but given its potential could be a promising therapeutic option.

Consistent with findings from human patients, studies from rodent models of TBI also report elevated brain expression of inflammasome component proteins [40]. Studies utilizing these animal models have predominantly assessed constitutive roles of inflammasome proteins by gene knockout methods, longitudinal expression of inflammasome and associated proteins after TBI and the effects of pharmacological intervention on disease outcomes. Surprisingly, in a CCI-induced mouse TBI model, genetic ablation of NLRP3 was shown to exaggerate astrocytic response and cytokine expression revealing the dual nature of NLRP3 signaling in acute neuroinflammation [45]. In contrast, NLRP1 inflammasome may not be relevant to outcomes in the mouse CCI model because gene deletion of NLRP1 or ASC did not influence functional recovery or contusion volume [27]. Therapeutic interventions to target the elevated inflammasome signaling are categorized as inflammasome-associated molecule inhibitors, specific pharmacological inhibitors, natural compounds, repurposed drugs and biologics (stem cell-derived vesicles/exosomes). The outcomes of these studies are summarized in Table 1. Unlike specific inhibitors, the mechanism for indirect inhibitors via natural products and repurposed drugs is largely unknown. These may be possibly related to the suppression of inflammasome activating signals [46] or the induction of negative regulators [47]. A novel approach of using biologics such as extracellular vesicles derived from stem cells has also been explored for targeting inflammasome activation after TBI. In a recent study, extracellular vesicles were isolated from human mesenchymal stem cell culture (hMSC-EVs) and validated for enrichment with microglia modulating miRNAs. Intranasal delivery of these hMSC-EVs in the CCI mouse model resulted in both acute and long-term suppression of the NLRP3 inflammasome, as well as improvements in long-term mood and cognitive deficits [48]. In a separate study, intravenous (IV) administration of adipose-derived stem cell exosomes (ADSCs-Exo) was found to inhibit NLRP3 inflammasome activity and improve sensorimotor function in a weight drop model of rat TBI [49]. These studies demonstrate the potential of such emerging biologic therapies for future exploration, for example in PTE research. However, a causal relationship is difficult to establish as they may act through different direct mechanisms and alterations in the inflammasome pathway may be a by-product of such outcomes, but they do provide additional associative evidence.

Overall, the outcomes from these studies consistently demonstrate that decreasing inflammasome and component protein expression in the brain after injury reduces cortical lesion size, which is associated with decreased inflammatory cytokine expression, and improvements in neurobehavioral and motor outcomes. However, none have explored post-traumatic epilepsy as an outcome measure in these studies. A recent review discussed in detail the specific inhibition of NLRP3 inflammasome in TBI and the compounds that are promising candidates for therapeutic targeting of the NLRP3 inflammasome in this context [40].

**Table 1:** Inflammasome expression/activity from clinical and preclinical TBI studies along with specific and non-specific NLRP3 inhibitors used in preclinical TBI studies

Clinical studies - TBI				
Subjects	Tissue/Fluid	Observations		Reference
Adults with moderate and severe TBI	CSF	Upregulated NLRP1, ASC and caspase-1 proteins.		[41]
Adults with moderate and severe TBI	CSF and serum	Elevated caspase-1 levels in serum on day 2 and 3 post TBI		[43]
Adults with severe TBI	CSF	Upregulated NLRP1, ASC and caspase-1 proteins.		[42]
Children with severe TBI	CSF	Increased NLRP3 levels at 0–24, 25–48, 49–72, and > 72 h post-TBI		[44]
Adults with severe TBI	Resected cortex	Upregulation of NLRP3, caspase-1, IL-1β, and IL-18 proteins		[47]
**Preclinical studies - TBI**				
**Inhibitor**	**Model, species**	**Effect on inflammasomes**	**Outcomes**	**Reference**
BAY 11-7082 (20 mg/kg IP) Once daily for 4 D.P.T	CCI, rats	Decreased protein levels of NLRP3 and caspase-1 in brain at 7 D.P.T.	Improved cognitive function and neuron viability at 7 D.P.T.	[50]
Anti ASC antibodies (20 µg/kg ICV) immediately P.T.	FPI, rats	Decreased protein levels of caspase-1, IL-1β and NLRP1 at 24 H. P.T.	Reduced contusion volume at 3 D.P.T.	[51]
MCC950 (50 mg/kg IP) 1- and 3-hours P.T.	CCI, mice	Decreased brain protein expression of NLRP3 ASC caspase-1and IL-1β at 24 H. P.T.	Decreased cerebral oedema and improved neurological function at 24 and 72 H P.T.	[52]
JC124 (100 mg/kg IP) 0.5, 6, 24 and 30 H.P.T.	CCI, rats	Decreased protein expression of NLRP3, ASC and caspase-1 in injured brain at 2 D.P.T.	Prevented neurodegeneration and decreased lesion volume at 2 D.P.T.	[53]
Mangiferin (100 mg/kg IP) 20 min P.T.	Blast-injury, rats	Decreased mRNA and protein levels of NLRP3 and caspase-1 p20 in brain at 12 and 24 H P.T.	Ameliorated cerebral oedema and oxidative stress at 12 and 24 H.P.T.	[46]
Omega-3 fatty acids (100 mg/kg oral gavage) twice a week for 6 weeks before TBI	CCI, rats	Decreased protein expression of NLRP3, caspase-1 and IL-1β in the brain at 8 H. P.T.	Decreased oedema and cortical lesion volume at 3 D and improved motor and cognitive performance at 11–15-D. P.T.	[47]
Apocynin (5 mg/kg IP) Every 24 h for 4 D.P.T.	CCI, rats	Decreased protein expression of NLRP3, ASC, caspase-1 and IL-1β in the brain at 4 D. P.T.	Decreased lesion size and neuronal death in injured cortex at 4 D. P.T.	[54]
Oridonin (10 mg/kg IP) 30 min P.T.	Weight Drop, mice	Decreased brain mRNA and protein levels of NLRP3, ASC and caspase-1 at 24 H. P.T.	Decreased oedema and cortical lesion volume at 24 H P.T, improved sensory motor function (2,4,7,14 D.P.T)	[55]
Dexmedetomidine (25 µg/kg IP for 3 D P.T)	CCI, mice	Decreased protein expression of NLRP3 caspase-1 and IL-1β in brain at 3 D.P.T.	Attenuated BBB disruption and neurological deficit at 3 D.P.T.	[50, 56]
Artesunate (30 mg/kg IP) 1 H.P.T.	CCI, mice	Decreased protein expression of NLRP3, ASC and caspase-1 in brain at 24 H. P.T.	Decreased lesion volume, inhibited apoptosis, and increased neurotrophic factors at 24 H. P.T.	[57]
Propofol (50 mg/kg IP) 1 H.P.T.	Blast injury, rats	Decreased brain mRNA and protein levels of NLRP3 and caspase-1 p20 at 12 H and 24 H P.T.	Attenuated cortical damage and oxidative stress at 12 H and 24 H P.T.	[58]
Pioglitazone (20 mg/kg IP) Daily till sacrifice (1,3,7,14 days)	Weight drop, mice	Decreased brain protein expression of NLRP3 on (3,7 D) caspase-1(1,3,7,14 D) and IL-1β 14 D. P. T	Reduced cerebral oedema and microglial activation at 3 D. P.T.	[59]
Telmisartan (10 mg/kg PO) 1 h before TBI	Cryogenic injury, mice	Decreased brain mRNA and protein levels of NLRP3, ASC and caspase-1 at 24 H P.T.	Reduced BBB disruption, decreased lesion volume, and improved neurological function at 24 H P.T.	[60]
hMSC-EVs Intra nasal, 90 min P.T.	CCI, mice	Decreased protein expression of NLRP3, ASC, caspase-1 and IL-1β in brain at 84 D.P.T.	Improved long term mood and cognitive impairment at 63–84 D.P.T.	[48]
ADSCs-Exo (IV, 24 h before TBI)	Weight drop, rats	Decreased protein expression of NLRP3 and caspase-1 at 24 H P.T.	Improved sensorimotor function at 7–35 D. P. T	[49]

CCI- Controlled Cortical Impact; FPI - Fluid Percussion Injury; hMSC-EVs- human mesenchymal stem cell culture derived extracellular vesicles; ADSCs-Exo- adipose-derived stem cell exosomes, D.P.T- Days Post TBI, H.P.T- Hours Post TBI, IP- Intra Peritoneal, IV- Intra Venous, ICV- Intra cerebroventricular, PO- Oral gavage

## Inflammasome signaling in epilepsy: evidence and therapeutic targeting

Chronic dysregulated inflammation has been heavily implicated in the pathogenesis of acquired focal epilepsy [61, 62]. More recently, specific investigations into inflammasome signaling have been conducted, and their impact on epileptogenesis assessed. Despite sufficient literature evidence implicating inflammasome activation in TBI progression and neurobehavioral outcomes, the role of inflammasome signalling in animal models of PTE has not been investigated so far and the research to date is mainly focused on preclinical models of acquired epilepsy. Therefore, we have discussed the pharmacological inhibition of inflammasomes in preclinical epilepsy models other than PTE and it remains to be investigated whether the same results in terms of seizure burden are achieved in animal models of PTE. While most clinical and preclinical studies of animal models report increased inflammasome expression which correlates with disease burden, animal studies have also revealed functional improvement after pharmacological inhibition of inflammasomes. The clinical and preclinical evidence has been described in detail as below (See Table 2).

**Table 2:** Inflammasome expression/activity from clinical and preclinical epilepsy studies. Autopsy brain tissues from non-diseased individuals were used as controls in clinical studies while healthy animals were used as controls in preclinical studies respectively. PTZ - Pentylenetetrazol, SE - status epilepticus

Clinical studies - Epilepsy				
Epilepsy type	Tissue	Outcomes		Reference
Temporal lobe epilepsy	Hippocampus	Upregulated NLRP1 and caspase-1		[63]
Temporal lobe epilepsy	Hippocampus	Upregulated NLRP3 and NLRP1 inflammasomes		[64]
Temporal lobe epilepsy	Hippocampus	Upregulation of NLRP1 inflammasome		[66]
Temporal lobe epilepsy	Temporal cortex	Upregulation of NLRP3 inflammasome		[65]
i) Focal epilepsy of unknown cause ii) Temporal lobe epilepsy	Blood	Suppressed or unchanged inflammasome activity		[68]
Epileptic patients with partial seizures	Blood	Correlation between NLRP1 polymorphism and partial seizures		[67]
**Preclinical studies - Epilepsy**				
**Model**	**Intervention**	**Effect on inflammasome**	**Outcomes**	**Reference**
**Inflammasome gene knockdown**				
Amygdala-kindling induced SE rats	NLRP1 and caspase-1 knockdown with siRNA infusion by osmotic pump for 6 weeks	Decreased protein expression of NLRP1, caspase-1 and IL-1β in brain at 6 weeks after SE.	Attenuated hippocampal neuronal pyroptosis and reduced seizure frequency and severity in chronic period at 6 weeks after SE.	[63]
Amygdala-kindling induced SE rats	NLRP3 and caspase-1 knockdown with siRNA infusion by osmotic pump for 6 weeks	Decreased protein expression of NLRP3, caspase-1 and IL-1β in brain at 6 weeks after SE.	Reduced hippocampal neuronal loss and decreased seizure severity and frequency at 6 weeks after SE.	[69]
**Natural compounds**				
PTZ induced kindling mice	Amentoflavone (25 mg/kg, PO) Alternate day for 29 days.	Decreased mRNA and protein levels of NLRP3, ASC and caspase-1 in brain at 24 h after last amentoflavone injection.	Reduced seizure frequency and duration, minimized cognitive dysfunction, and blocked neuronal apoptosis at 24 h after last amentoflavone injection.	[70]
PTZ induced kindling mice	Semaglutide (L.D- 10 nM/kg, H.D- 25 nM/kg IP) Alternate day for 37 days, last on 45th day.	Decreased mRNA and protein expression of NLRP3, ASC and caspase-1 in brain at one week after last Semaglutide injection.	Reduced seizure severity and ameliorated cognitive dysfunction at one week after last Semaglutide injection	[71]
PTZ induced kindling in mice	Agmatine (10 mg/kg, IP) Alternate day for 28 days.	Decreased brin mRNA and protein levels of NLRP3, ASC and caspase-1 after treatment end (28 days)	Reduced seizure incidence and duration (from day 14th ), alleviated hippocampal neuronal damage after treatment end (28 days).	[72]
**NLRP3 specific inhibitors**				
PTZ induced kindling in mice	CY-09 (2.5 mg/kg, IP) Alternate day for 22 days	Decreased the levels of caspase-1 and IL-1β in brain at 24 h after last CY-09 injection.	Suppressed seizure severity during the treatment and attenuated hippocampal neuronal loss after treatment end.	[73]
Kainic acid-induced SE in mice	MCC950 (10 mg/kg, IP) Daily for 7 days	Decreased the levels of caspase-1 and IL-1β in brain after treatment end.	Reduced hippocampal neuronal loss after treatment end.	[74]

IP- Intra Peritoneal, IV- Intra Venous, ICV- Intra cerebroventricular, PO- Oral gavage

### Clinical evidence

Elevated protein levels of NLRP1, NLRP3 and active caspase-1 have been identified in sclerotic hippocampi of patients with temporal lobe epilepsy when compared to the healthy non-epileptic control tissues obtained from biobanks or patients with other disorders [63, 64]. In another study, NLRP3 inflammasome levels were upregulated in the temporal cortices of patients with temporal lobe epilepsy (TLE) and associated with endoplasmic reticulum stress (ERS) [65]. This indicates possible crosstalk between inflammasome signaling and other neuroinflammatory mechanisms such as ERS in epilepsy. Similar alterations in the inflammasome signaling have been reported at transcriptome levels, with increased NLRP1 levels observed in the resected hippocampi of TLE patients with hippocampal sclerosis highlighting a possible role of NLRP1 in TLE pathogenesis [66]. In a study investigating in Chinese patients, NLRP1 polymorphism was found to be associated with an increased risk of epilepsy development. In this study, it was shown that the seizure frequency in recruited patients correlated with the polymorphism in NLRP1 inflammasome genes [67].

Contrary to other reports of inflammasome signaling in the brain, a recent study reported a suppressed inflammasome expression in CD3 + and CD14 + peripheral blood mononuclear cells (PBMC) in patients with mesial temporal lobe epilepsy and focal epilepsy of unknown cause. This study evaluated the inflammasome signaling in peripheral immune cells as compared to the evaluation of brain levels and suggests that the brain levels of inflammasome components may not display a surrogate marker in the peripheral immune cells, at least in epilepsy patients [68].

### Preclinical evidence with therapeutic targeting of inflammasomes

Studies utilizing animal models of epilepsy have shown beneficial outcomes following therapeutic inhibition of NLRP3 inflammasomes. The first study to report the involvement of inflammasomes in epilepsy was performed in a rat model of status epilepticus (SE). SE was induced by the electrical stimulation of the amygdala, which led to significant upregulation of NLRP3, caspase-1, and IL-1β in the hippocampus of stimulated animals at 3 h and peaked at 12 h post-SE. Importantly, siRNA-mediated knockdown of NLRP3 or caspase-1 was able to decrease the levels of IL-1β and this was associated with a reduction in frequency and severity of epileptic seizures and hippocampal cell loss [69]. In another study by this group, siRNA-silencing of NLRP1 and caspase-1 in an amygdala kindling-induced rat TLE model revealed a significant reduction in neuronal pyroptosis along with a reduction in the frequency and severity of epileptic seizures [63]. Activation of the NLRP3 inflammasome is also seen after SE caused by chemo-convulsant. Improved NLRP3 inflammasome activation has been shown by Zhang et al. to hasten the course of SE in a rat model of kainic acid-induced SE. In this study, the direct inhibitor MCC950 substantially attenuated the increased levels of inflammatory proteins and decreased hippocampal neuron death by inhibiting NLRP3 [74]. MCC950 selectively inhibits NLRP3 by binding directly to the NACHT domain of the NLRP3 protein [75]. Similarly, in the pentylenetetrazol (PTZ) kindling model, seizure severity was decreased, and hippocampal neuronal apoptosis attenuated after treatment with a specific NLRP3 inhibitor CY-09 [73].

In addition to specific inhibition of NLRP3 inflammasome proteins, some of the compounds with antioxidant activity have also shown the potential to alter inflammasome signaling in epilepsy models. In two separate studies utilizing the PTZ-induced kindling mouse model, treatment with amentoflavone, a natural bioflavonoid compound, and Semaglutide, a type II antidiabetic drug, was shown to delay PTZ kindling progression along with reduced hippocampal neuronal apoptosis and an improvement in spatial learning. The hippocampal mRNA and protein levels of NLRP3, ASC and caspase-1 were significantly reduced in drug-treated mice as compared to control animals indicating that the neuronal and functional deficits post PTZ kindling could be mediated via NLRP3 inflammasome activation [70, 71]. In a similar study, administration of Agmatine, an endogenous amine synthesized from the decarboxylation of L-arginine, was also reported to decrease seizure susceptibility and delayed latency and duration of generalized seizures in the PTZ kindling model [72]. In all the above-mentioned studies utilizing natural compounds, the authors investigated the impact of drug treatment on lipopolysaccharide-induced BV2 microglial cells and showed that it led to the suppression of inflammasome pathway proteins, which supports the in-vivo findings and the role of NLRP3 inflammasome pathway in epileptogenesis [70–72].

Evidence for inhibition of caspase-1 has also been reported to show beneficial outcomes in animal models of epilepsy. In a mouse model of acute seizures induced by unilateral intrahippocampal kainic acid injection, systemic administration of VX-765, a caspase-1 inhibitor, delayed the onset of seizures and reduced the frequency of convulsive episodes. The same study also reported chronic outcomes following induction of SE using unilateral intrahippocampal kainic acid injection and observed dose-dependent and reversible reduction in spontaneous epileptic activity [76]. Taken together, all these findings indicate that the inflammasome may represent a promising therapeutic target for the treatment of acquired epilepsy. As discussed previously, the impact of some of the antioxidant/natural compounds on inflammasome signaling may be an associative finding or a consequence of the suppression of epileptic seizures with unrelated mechanisms. Therefore, a causal relationship between these compounds and inflammasome signaling remains evasive.

## Downstream mediators of inflammasome signaling in epileptogenesis

**Fig. 2:**
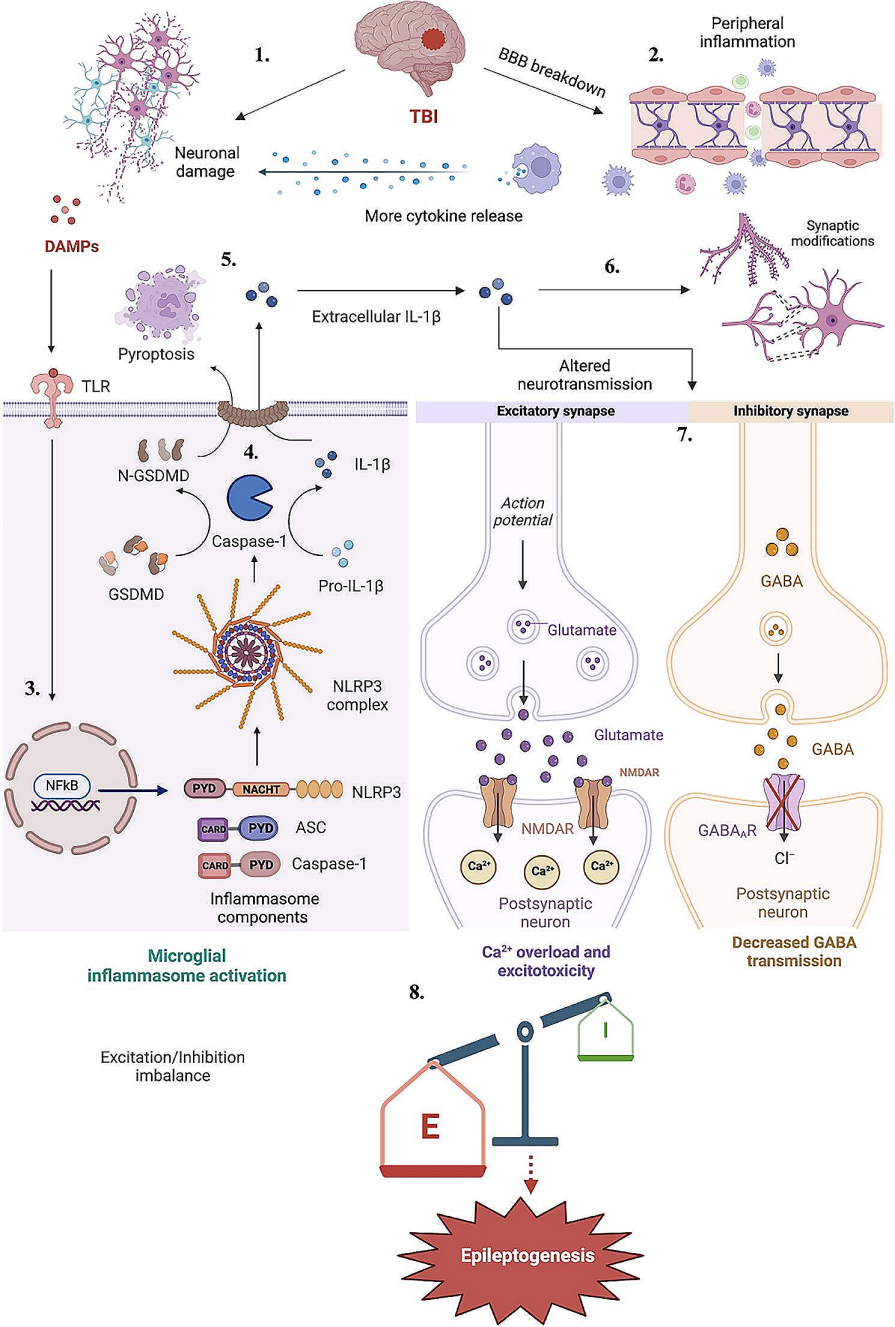
Mechanisms by which NLRP3 inflammasome activation increases the susceptibility to post-traumatic epileptogenesis. (1) TBI causes neuronal damage resulting in DAMP generation. (2) TBI also causes BBB breakdown resulting in infiltration of peripheral inflammatory cells causing cytokine release and further neuronal damage. (3) DAMPs initiate TLR-mediated NFκB activation and subsequent expression of the inflammasome genes within microglia. (4) Synthesis of mature IL-1β and Gasdermin D by activated caspase-1. (5) Plasma membrane rupture causing pyroptotic cell death by Gasdermin D and release of IL-1β in extracellular space.6) Reduced synaptic density and abnormal synapse formation 7) Alteration of NMDA and GABA receptors by extracellular IL-1β 8) Imbalance between excitation and inhibition ultimately contributing to epileptogenesis. BBB- blood-brain barrier, DAMP- Damage associated molecular patterns, NMDAR- N-methyl-D-aspartate receptor, AMPAR- α-amino-3-hydroxy-5-methyl-4-isoxazole propionic acid receptor, GABAR- Gamma-aminobutyric acid receptor, GSDMD- Gasdermin D, IL-1β - Interleukin-1β

Having listed evidence for the involvement of inflammasome signaling in epilepsy, we next explored how the downstream signaling partners which can be activated by inflammasome signaling may directly impact epileptogenesis. In this regard, several potential mechanisms may link the effector molecules of inflammasome activation, such as caspase-1, Gasdermin and cytokines including IL-1β and IL-18, to cellular hyperexcitability and, hence, epileptogenesis (summarized in Fig. 2).

### Caspase-1

Conventionally, caspase-1 and other cysteine proteases have largely been studied in the context of apoptotic pathways [77]. After the discovery of inflammasomes as caspase-1 activating platforms, the focus shifted to exploring the contribution of caspases in various CNS pathologies. As a key component of inflammasome signaling, caspase-1 is well placed to influence post-traumatic epilepsy through the activation of several downstream products, including, for example, IL-1β, Gasdermin D, and IL-18 another pro-inflammatory cytokine released from a variety of cells such as monocytes, macrophages, and keratinocytes [78]. Some evidence also directly implicates this enzyme [79]. For example, initial preclinical studies reported overexpression of caspase-1 in epileptic conditions and proposed the inhibition of caspase-1 as a novel anticonvulsant strategy [80]. Subsequent studies reported the ability of caspase-1 to confer pharmaco-resistance in animal models of epilepsy, though the exact molecular mechanism remains unknown [81]. Furthermore, caspase-1 appears necessary for certain seizure types such as febrile seizures, since mice lacking caspase-1 were found to be resistant to febrile seizure induction [82]. Despite exploring the therapeutic potential of targeting caspases in neurodegenerative diseases including epilepsy, a lack of understanding of the basic functions of different caspases has hampered progress towards clinical trials [83, 84].

### IL-1β signaling

One of the best characterized and most studied of the IL-1 family members, IL-1β, is produced and secreted by innate immune cells like monocytes, macrophages in the periphery and microglia in the brain. As mentioned in the earlier section, caspase-1 mediates the cleavage of precursor pro-IL-1β downstream of inflammasome activation. Though caspase-1 mediated activation is almost exclusive, IL-1β processing is also carried out by proteases derived from neutrophils or invading pathogens [85]. The matured IL-1β then binds with IL-1R and initiates the transcriptional activation of NF-κB and MAPK, causing enhanced induction of cytokine and chemokine synthesis. Additionally, alteration of BBB permeability, recruitment of peripheral inflammatory cells and oedema formation have also been reported as extracellular actions of IL-1β post-TBI [86]. One of the direct mechanisms of IL-1β-mediated alteration of BBB permeability was shown to be the enzymatic degradation of endothelial tight junction proteins in a study conducted in a mouse CCI model of TBI [87].

The expression pattern of IL-1β over time may suggest differential roles in acute and chronic TBI pathophysiology. In a preclinical mild closed head injury model, transient upregulation of IL-1β was found to drive the acute neuroinflammatory response in the early hours after traumatic brain injury (TBI), while persistent IL-1β levels were responsible for the behavioural impairment seen at 14 weeks post-injury [88].

As for epilepsy development, chronic upregulation of IL-1β can influence both excitatory and inhibitory neurotransmission, resulting in hyperexcitability and decreased seizure threshold [89]. This occurs primarily due to post-translational modifications of receptor channels such as phosphorylation of the NR2A/B subunits of the NMDA receptor, and subsequent increase in calcium influx which may lead to neuronal hyperexcitability and cell death [90]. In addition, increased glutamate discharge from glial cells and decreased astrocytic glutamate uptake are also attributed to IL-1β activity [91, 92], an effect mediated by the downregulation of extracellular amino acid transporters [93], which may result in excessive excitatory neurotransmission. IL-1β has also been shown to interfere with GABAergic neurotransmission. In a study conducted on hippocampal tissue of TLE patients, and in rat models of acquired epilepsy, IL-1β treatment decreased the amplitude of GABA-evoked currents [94]. This IL-1β-induced decrease in GABAergic transmission has also been linked with decreased neuronal Cl^−^ influx, as observed in cultured hippocampal neurons [95].

Apart from the altered neurotransmission, IL-1β has been directly linked with synaptic loss through mechanisms such as NMDA receptor-mediated ubiquitin-proteasome pathway and upregulated P-38/MAPK/COX 2 pathway in rat hippocampal neuronal culture [96]. Moreover, in cultured rat cortical neurons, IL-1β lowered synaptophysin synthesis via the P-38/MAPK pathway, indicating a potentially deleterious effect of IL-1β on synaptic proteins [97]. As previously mentioned in the article, activation of the NF-κB and/or P-38/MAPK pathway can sustain the neuroinflammatory cascade by upregulation of cytokines, including IL-1β [98].

The aforementioned cellular alterations wrought by IL-1β align with its effects on seizures. For example, preclinical studies have shown that intra-hippocampal injection of IL-1β increased acute seizure activity and lowered seizure threshold in rodents treated with convulsive drugs, which subsequently reduced after inhibition of the IL-1R1 signaling or caspase-1 blockade [76, 80]. Also, in a separate study, IL-1R1 antagonism was shown to reduce seizure susceptibility after pro-convulsant treatment in a mouse model of pediatric brain injury [99]. IL-1β can further induce microglia and astrocytes creating a positive feedback loop and ultimately evolving into a chronic unchecked neuroinflammatory response [93]. Overall, inflammasome-mediated activation of IL-1β is a strong candidate to influence epileptogenesis after TBI [100].

### Gasdermin D-mediated pyroptosis

Pyroptosis is a characteristic inflammatory cell death triggered by activated caspase-1 downstream of inflammasome activation. This pro-inflammatory nature of pyroptosis has recently been promoted as an important target in neurodegenerative disorders, including epilepsy [101]. It is mediated by a family of pore-forming proteins known as the Gasdermins, which are activated by caspase-1 [102]. Among the Gasdermins, Gasdermin D (GSDMD) remains the most well-studied. Pore formation by GSDMD triggers pyroptotic cell death through rupture of the plasma membrane and leakage of cytosolic contents [36, 103], stimulating phagocytosis [104]. The leakage of inflammatory cytokines, as well as the DAMPs, from dying cells, attracts further inflammatory mediators, damaging the surrounding cells [105]. In a rat model of TBI, GSDMD was significantly elevated in the injured cortex along with microglial localization. The same study showed that GSDMD knockout ameliorated neurological deficits and its cleavage was mainly driven by NLRP3 inflammasome [106]. Active pyroptosis has been demonstrated in the amygdala kindling-induced rat model, and components of pyroptosis identified in excised temporal lobe epilepsy specimens suggest this cell death pathway may be relevant for disease pathogenesis [63]. To further investigate the role of pyroptosis in epilepsy, a bioinformatics study was conducted to identify a gene set that was closely related to human epilepsy and pyroptosis respectively using a weighted gene co-expression network analysis. The authors observed a close relationship between the gene sets related to human epilepsy and the pyroptosis pathway. Furthermore, using a kainic acid-induced SE model in mice, they observed a significant upregulation of mRNA and protein levels of GSDMD in the hippocampus. Although these studies do not explain the exact molecular mechanism, the engagement of pyroptosis during epilepsy development was confirmed and authors suggested that it could be an important target for intervention [107].

Interestingly, alternate inflammatory pathways, such as cGAS-STING-mediated type-1 interferon (IFN) signaling have been shown to contribute towards the activation of the NLRP3 inflammasome and pyroptosis in a rat TBI model [108]. Other preclinical TBI studies reporting increased STING-mediated type-1 IFN signaling, reactive gliosis, and cytokine upregulation corroborate this finding, with the deficiency of the IFN a/b receptor effectively ameliorating microgliosis and neurobehavioral dysfunction [109, 110]. Similarly, transcriptomic analysis of ipsilateral cortical astrocytes of PTE mice showed the involvement of IFN signaling based on altered gene expression [111]. Together, these are suggestive of cGAS-STING and IFN-1 signaling as potential upstream regulators of NLRP3 and the crosstalk between different inflammatory pathways which may be relevant to PTE. In the future, a combinatory approach of utilizing molecular biology and bioinformatics tools can further our understanding of the contribution of these pathways in epilepsy and other neurodegenerative disorders [108].

## Inflammasome proteins as biomarkers of PTE

The discovery of valid predictive and diagnostic biomarkers has emerged as one of the crucial requirements in PTE research and is highlighted by the International League against Epilepsy [109]. Considering the far-reaching scope of neuroinflammation in the pathogenesis of TBI and PTE, proteins like inflammasomes which are an integral part of this process could be potential biomarkers of not only TBI progression but also of epileptogenesis. Some evidence already supports this potential.

Firstly, multiple clinical studies have reported elevated expression of inflammasome components and cytokines in serum and CSF of patients following TBI [41, 110, 111]. One study exploring the potential of inflammasome signaling as a predictive biomarker of TBI outcomes showed a higher level of ASC, caspase-1, and NALP-1 in the CSF of TBI patients that was collected within 72 h post-injury. The levels of these proteins correlated significantly to the worst outcomes based on the Glasgow outcome scale evaluated at 5-months post-TBI. The levels were also higher in patients with unfavorable outcomes, such as severe disability and death, 5-months post-TBI [41]. Recently, brain-derived extracellular vesicles (EV) containing inflammasome proteins have been shown to propagate inflammation after CNS injury [42]. In a stroke patient study, the expression of serum ASC following EV isolation was found to be a valid biomarker of stroke as determined by ROC analysis (AUC 1), suggesting the significant biomarker potential of inflammasome proteins and indicating their prospective application in other CNS disorders such as PTE [112].

Secondly, several investigations have demonstrated the involvement of inflammasome downstream signalling mediators, such as IL-1β, in post-traumatic consequences such as excitotoxicity and premature neuronal death, underscoring the possible link between IL-1β and the risk of PTE [113–115]. One study reported that individuals with a higher CSF/serum ratio of IL-1β one-week post-injury had an increased risk of developing PTE. Moreover, the same study reported that genetic polymorphisms of the IL-1β gene increased the susceptibility of PTE [113].

Also, apart from the conventional fluid biomarkers, imaging of neuroinflammation with modalities like Positron Emission Tomography (PET) imaging of translocator protein (TSPO) holds promise as biomarkers for PTE. TSPO is a protein located on the outer mitochondrial membrane and thought to be representative of inflammatory cell activation/density, has been used to assess neuroinflammation in several neurodegenerative disorders [116]. An increase in TSPO binding, indicative of enhanced neuroinflammation, is observed in TLE patients [117] and is predictive for seizure frequency following an epileptogenic insult in a rodent model of epilepsy (kainic acid-induced status epilepticus) [118]. Given the contribution of inflammasomes to neuroinflammation, there is understandable interest in developing radiotracers targeting inflammasome proteins such as NLRP3. For example, a recent study reported the synthesis of a carbon 11 labelled NLRP3 inhibitor *N-(5-Chloro-2-methoxybenzyl)-N-(4-(N-(prop-2-yn-1-yl)-sulfamoyl)phenethyl)-2-(thiophene-3-yl)acetamide*, which exhibited rapid BBB penetration and moderate and blockable brain uptake PET imaging in mice [119].

Though the development of inflammasome-specific tracers seems to be focused on optimizing the lead inflammasome inhibitor compounds for therapeutic purposes, future tracers targeting the component proteins of inflammasome assembly could provide some novel insight for predicting the development and outcome of PTE. The correlation of imaging data with fluid biomarker levels, behavioral comorbidities and seizure frequency and severity can further validate the biomarker potential of inflammasomes.

## Conclusions and future directions

PTE is a relatively common and disabling feature of TBI. While managing TBI is challenging, understanding the development and progression of PTE is much more elusive. The lack of effective therapeutic interventions has prompted the investigation of novel molecular pathways that lead to the development of PTE. Literature evidence has suggested that inflammasomes could be drivers of chronic neuroinflammation and potentially responsible for the worsening of TBI and initiating epileptogenesis.

Recent clinical and experimental findings have independently demonstrated the upregulation of inflammasome signaling in the pathogenesis following TBI, as well as their involvement in the development of seizures and epilepsy. Moreover, the downstream mediators of inflammasome signaling are strongly implicated in disease development, and inhibition of inflammasomes can improve outcomes of both conditions in experimental models. This suggests that inflammasomes may represent an important therapeutic target for managing neuroinflammation and improving patient outcomes, including the PTE following TBI. In addition, emerging reports suggest that inflammasome proteins and downstream signaling molecules may be useful as potential biomarkers of neuroinflammatory conditions following TBI and early prediction of post-traumatic complications. Given that inflammatory processes could also predict the risk or severity of epilepsy, it is hypothesized that inflammasome components upregulated after TBI could represent a potential biomarker of PTE. In the future, studies evaluating the therapeutic efficacy of manipulating inflammasome pathways for the development of post-traumatic after TBI need to be conducted. Furthermore, fluid and neuroimaging markers of inflammasome may have the potential to predict the risk of PTE and select patients most likely to benefit from anti-epileptogenic interventions. Their nature would also better understand direct molecular mechanisms linking inflammasome activation and epilepsy development. Through a combination of these strategies, the development of novel pharmacological therapies to modulate the inflammatory response post-TBI could be applied to the clinical subjects following TBI who are at high risk of developing PTE.

## Data Availability

No datasets were generated or analysed during the current study.
